# Methane-Oxidizing Bacteria Shunt Carbon to Microbial Mats at a Marine Hydrocarbon Seep

**DOI:** 10.3389/fmicb.2017.00186

**Published:** 2017-02-27

**Authors:** Blair G. Paul, Haibing Ding, Sarah C. Bagby, Matthias Y. Kellermann, Molly C. Redmond, Gary L. Andersen, David L. Valentine

**Affiliations:** ^1^Department of Earth Science, University of California, Santa Barbara, Santa BarbaraCA, USA; ^2^Marine Science Institute, University of California, Santa Barbara, Santa BarbaraCA, USA; ^3^Key Laboratory of Marine Chemistry Theory and Technology, Ministry of Education, Ocean University of ChinaQingdao, China; ^4^Earth Sciences Division, Lawrence Berkeley National Laboratory, BerkeleyCA, USA

**Keywords:** microbial mats, methanotrophs, sulfide-oxidizing bacteria, stable isotope probing, intact polar lipids (IPL), 16S rRNA gene

## Abstract

The marine subsurface is a reservoir of the greenhouse gas methane. While microorganisms living in water column and seafloor ecosystems are known to be a major sink limiting net methane transport from the marine subsurface to the atmosphere, few studies have assessed the flow of methane-derived carbon through the benthic mat communities that line the seafloor on the continental shelf where methane is emitted. We analyzed the abundance and isotope composition of fatty acids in microbial mats grown in the shallow Coal Oil Point seep field off Santa Barbara, CA, USA, where seep gas is a mixture of methane and CO_2_. We further used stable isotope probing (SIP) to track methane incorporation into mat biomass. We found evidence that multiple allochthonous substrates supported the rich growth of these mats, with notable contributions from bacterial methanotrophs and sulfur-oxidizers as well as eukaryotic phototrophs. Fatty acids characteristic of methanotrophs were shown to be abundant and ^13^C-enriched in SIP samples, and DNA-SIP identified members of the methanotrophic family Methylococcaceae as major ^13^CH_4_ consumers. Members of Sulfuricurvaceae, Sulfurospirillaceae, and Sulfurovumaceae are implicated in fixation of seep CO_2_. The mats’ autotrophs support a diverse assemblage of co-occurring bacteria and protozoa, with Methylophaga as key consumers of methane-derived organic matter. This study identifies the taxa contributing to the flow of seep-derived carbon through microbial mat biomass, revealing the bacterial and eukaryotic diversity of these remarkable ecosystems.

## Introduction

The oxidation of methane by marine microorganisms is a key control in the flux of this potent greenhouse gas from the ocean to the atmosphere (Reeburgh, 2007). Numerous bacterial phyla contribute to methane oxidation, including both the methanotrophs that metabolize methane directly and the non-methanotrophic methylotrophs that use methanol and other partially oxidized methane metabolites (Hanson and Hanson, 1996). In the presence of hydrocarbons, methanotrophic activity can support heterotrophic bacteria and eukaryotes in a range of marine communities (Childress et al., 1986; Cavanaugh et al., 1987; Hanson and Hanson, 1996). Depending on light intensity and substrate availability, these communities may also have access to primary production via photoautotrophy and chemoautotrophy.

A range of niches within benthic microbial mat communities can allow a broad array of organisms—chemoautotrophs, photoautotrophs, and heterotrophs alike—to play critical roles in nutrient cycling (Stanish et al., 2013; Scott et al., 2015; Stuart et al., 2015; Valentine et al., 2016). Marine microbial mats are typically filamentous and dominated by sulfide-oxidizing bacteria, including Beggiatoa, Thioploca, Sulfurovum, and Sulfurimonas (Nelson et al., 1989; Zhang C.L. et al., 2005; Moussard et al., 2006; Gilhooly et al., 2007). These communities may support thriving populations of benthic grazers and other fauna (Van Dover and Fry, 1994; Al-Zaidan et al., 2006).

To date, characterization of methane seep microbial mats has been limited to seeps occurring in dark or low-oxygen waters. Cold seeps in the anoxic Crimea shelf of the Black Sea support mat communities dominated by anaerobic methanotrophs (Blumenberg et al., 2005; Knittel et al., 2005; Treude et al., 2005). Similarly, methanotrophy is primarily anaerobic in mats supported by mixed gas and oil seeps at 3000 m in the Gulf of Mexico (Schubotz et al., 2011). Aerobic and anaerobic methanotrophs co-occur in the sulfide-oxidizing microbial mats of the Haakon Mosby Mud Volcano (HMMV; Niemann et al., 2006; Lösekann et al., 2007), which grow without light and at low ambient oxygen at 1250 m water depth. Currently, our understanding of cold seep mat communities from shallow, oxygen-rich continental shelf environments appears limited (Ruff et al., 2015).

A shallow, oxygen-rich hydrocarbon seep occurs at Coal Oil Point (COP), California, covering >4200 m^2^ (Washburn et al., 2005). In terms of total gas flux, it is one of the largest hydrocarbon seep fields in the world (Hornafius et al., 1999). A single seep location in particular, known as Shane Seep, lies at 22 m water depth within COP and emits up to 3300 m^3^ of gas per day (Clark et al., 2003; Washburn et al., 2005). Previous ecological studies at the COP seeps have demonstrated that microbial abundance increases in close proximity to actively venting hydrocarbon seeps (Montagna et al., 1987; Ding and Valentine, 2008), providing a distinctive setting with respect to deeper hydrocarbon seeps because of strong seasonality and the availability of sunlight to drive photosynthesis. In turn, meiofaunal and macrofaunal communities appear to be supported by microbial mats at the seeps (Montagna et al., 1987). While the microbial communities within COP sediments have been studied (LaMontagne et al., 2004; Redmond et al., 2010), the abundant benthic mats of this seep field have gone largely unstudied, beyond suggestive lipid evidence for methanotrophy (Ding and Valentine, 2008). The community composition of these mats remains entirely uncharacterized. The obvious differences in irradiance, turbulence, and the physical matrix supporting growth between COP mats and deep ecosystems led us to predict that community structure would differ between shallow and deep seeps.

We set out to determine the community composition of microbial mats growing at Shane Seep, and to establish whether these mats contribute to methane oxidation. We made use of the fact that biomass of primary producers carries the isotopic signature of their carbon source. *Ex situ*, we performed stable isotope probing (SIP), incubating mats with ^13^C-labeled methane. We followed this tracer into two pools of biomass, measuring the isotope signatures of individual lipids and the redistribution of DNA from individual taxa across a density gradient. *In situ*, the natural ^13^C-depletion of seep methane and ^13^C-enrichment of seep CO_2_ allowed us to track these carbon sources into mat community biomass, and to identify the taxa contributing most heavily to seep gas uptake. Finally, looking beyond methane, we asked which other ecological roles were represented in the total community. Collectively, the fatty acid, intact polar lipid (IPL), 16S rRNA gene, and 18S rRNA gene profiles we obtained offer a multifaceted characterization of this ecologically interesting microbial community.

## Materials and Methods

### Study Site and Growth Surface

**FIGURE 1:**
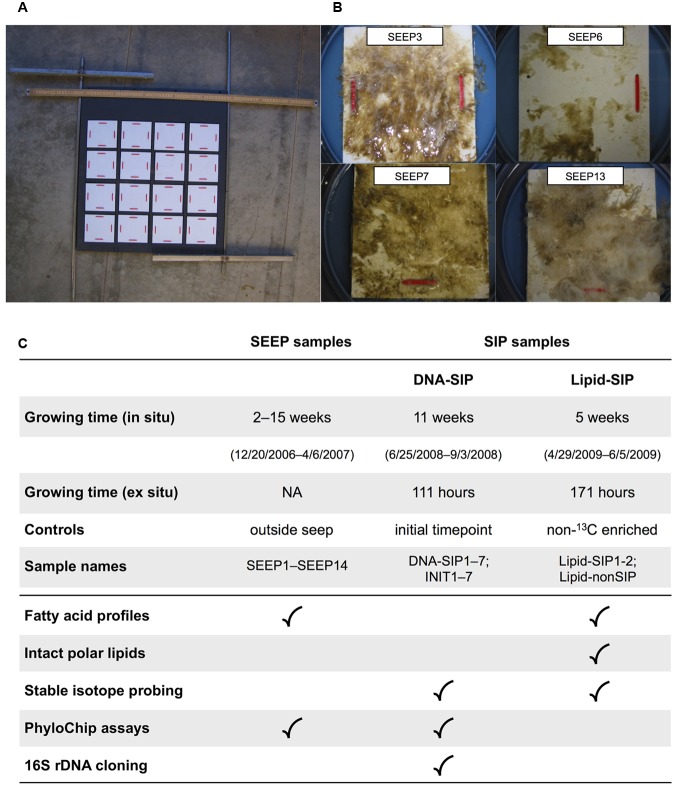
**Summary of growth and enrichment experiments: (A)** Benthic growth device. Rough PVC growth plates (white) were affixed to a large mounting board facing down when deployed, and secured *in situ* with rebar driven into the seafloor. Individual growth plates were removed by SCUBA divers, leaving the remaining plates undisturbed. For scale, a metric measuring stick is shown at the top of the mounting board. **(B)** Representative growth plates recovered from Shane Seep during *in situ* sampling. **(C)** Overview of sampling and analyses performed on environmental (SEEP) and experimental (SIP) mats.

Shane Seep lies in the Coal Oil Point seep field offshore Goleta, CA (**Table 1**) at 22 m water depth and emits a continuous flux of gas. The seep gas, a mixture of CO_2_ and short-chain hydrocarbons, has been the subject of several geochemical studies (Clark et al., 2003; Washburn et al., 2005; Kinnaman, 2008). Microbial mats grow directly on the seafloor. While these mats can be harvested by scraping, this process disrupts mat integrity and viability for *ex situ* incubations. Instead, we deployed a 60 cm × 60 cm *in situ* colonization assembly (**Figure 1A**) that provided a rough PVC surface for mat growth and enabled minimally disruptive removal of 10 cm × 10 cm sections of mat for analysis.

**Table 1:** Summarized metadata for Shane Seep.

Seep Parameters							
**Location**	**Depth (m)**	**Mean Temperature (°C)**	**Gas Flux (m^3^ day^-1^)^1^**	**%CH_4_^2^**	**%CO_2_^2^**	**δ^13^C-CH_4_ (‰)^2^**	**δ^13^C-CO_2_ (‰)^2^**
34°, 24.370′N;	22	12.6	3300	81.0–85.7	12.0–16.7	-50.5 to -53.9	15.8–18.4
119°, 53.428′W							

*^1^Reported by Washburn et al. (2005).*

*^2^Percent of total hydrocarbon and CO_2_ gas, measured by Kinnaman (2008)*

*Samples collected from 21 m depth.*

### SEEP Sampling

In December 2006, the colonization assembly was deployed over an active gas vent at Shane Seep (**Figure 1A**). A duplicate growth device was positioned 30 m from the seep field, with the intent of providing biomass to compare with Shane Seep mat biomass. Following deployment, mats were allowed to form and grow without sampling for 2 weeks, after which SCUBA divers retrieved one 10 cm × 10 cm mat section each week (**Figure 1B**) for chemical and lipid analysis. These were designated SEEP samples. Samples were transported to the laboratory in sealed containers with seawater collected *in situ*. Mats were held at *in situ* temperature (12°C) and processed for analysis on the same day (see below). After 14 weeks of sample retrievals, a large offshore storm with strong wave action mangled the deployed growth devices (Supplementary Figure S1), forcing an end to SEEP sampling in April 2007.

### SEEP Elemental and Fatty Acid Analyses

The elemental composition (wt % C, H, N, and S) of SEEP mats was determined using a CEC 440HA automated organic elemental analyzer (Exeter Analytical). Mats were subsampled for fatty acid extraction following the method of Ding and Sun (Ding and Sun, 2005). Briefly, mat subsamples were scraped from growth plates and extracted with methanol and methylene chloride:methanol (2:1 v/v). Following saponification in 0.5 M KOH/methanol and re-extraction in hexane at pH 1–2 (pH adjusted with 6 M HCl), fatty acids were heated with a solution of BF3 in methanol to form fatty acid methyl esters (FAMEs). FAMEs were separated using an HP-5890 series II gas chromatograph (GC) with a 30 m × 0.25 mm AT^TM^-5MS capillary column (Alltech) and flame ionization detector. Concentrations were determined with an HP-3396 series III integrator and peaks were compared to the internal standard nonadecanoic acid methyl ester, as previously described (Ding and Valentine, 2008).

Stable carbon isotope ratios (δ^13^C) were measured by isotope ratio mass spectrometry (IRMS), using a HP 5970 GC mass selective detector and Delta Plus XP Mass Spectrometer (Thermo Finnigan) at the UC Santa Barbara Marine Science Institute Analytical Laboratory, as previously described (Ding and Valentine, 2008). Briefly, FAME compounds were separated by gas chromatography (GC) using nonadecanoic acid methyl ester as a standard, with helium carrier gas at a flow rate of 1.0 mL/min. FAME compounds were oxidized to CO_2_ with Cu/Ni/Pt wire at 950°C *via* a GC combustion interface and measured by IRMS relative to a CO_2_ standard (Air Liquid), corrected for the isotope ratio of the added methyl group.

### Scanning Electron Microscopy and X-ray Spectrometry

Scanning electron microscopy (SEM) and X-ray spectrometry was conducted as previously described (Sun et al., 2016). Briefly, mat subsamples were frozen at −80°C for 4 h, then freeze-dried with an ALPHA 1-2 LD plus freeze-dryer (GmbH, Germany). Next, samples were placed on conductive carbon tabs with SEM posts and a Hitachi E-1045 coater was used for gold sputter-coating (Hitachi High-Tech Science Corp., Japan). The prepared samples were viewed using a Hitachi S-4800 emission scanning electron microscope and analyzed using a HORIBA 7593-H energy-dispersive X-ray spectrometer (Horiba Ltd., UK). Energy-dispersive X-ray spectrometry was conducted with the following parameters: 15 mm distance; 300 s data acquisition time; 2000 speed.

### SEEP DNA Analyses

We analyzed the 16S rRNA gene-based taxonomic structure from eight environmental SEEP samples (SEEP 1, 3, 4, 6, 7, 9, 11, and 13) with high, low, and intermediate total δ^13^C values of key fatty acids. In addition, we cloned and sequenced 18S rRNA genes to profile the eukaryotic community in one sample (SEEP 3). DNA was extracted from microbial mats using a bead beating and spin column protocol (Fast DNA SPIN for Soil, MP Biomedicals). PCR amplification of 16S and 18S rRNA genes was conducted with the following thermal cycling conditions: initial denaturation at 95°C for 3 min; 30 cycles of 92°C denaturation for 1 min, 55°C annealing for 1 min, 72°C extension for 1 min; final extension at 72°C for 5 min. PCR reactions used universal primers for bacteria: 27F, 5′-AGAGTTTGATCCTGGCTCAG-3′ and 1492R 5′- GGTTACCTTGTTACGACTT-3′ (Lane, 1991; Redmond et al., 2010) and eukaryotes: 515F, 5′-GTGCCAAGCAGCCGCGGTAA-3′ and 1209R 5′-GGGCATCACAGACCTG-3′ (Baker et al., 2009). PCR products were cleaned using the SV Wizard PCR Cleanup Kit (Promega) and quantified using a BioAnalyzer 2100, with high sensitivity dsDNA reagent kits (Agilent Biosciences).

High-density 16S rRNA gene G2 microarrays (Brodie et al., 2007) were used to examine the bacterial community structure of SEEP samples. The G2 PhyloChip uses ∼300,000 sequence probes to assay microbial diversity at the sub-family and OTU level. PhyloChip processing was conducted at Lawrence Berkeley National Laboratory (LBNL) as previously described (Brodie et al., 2007). Briefly, 16S rRNA gene PCR product (500 ng) was spiked with an internal standard, then fragmented using DNase; fragments were biotinylated and hybridized (48°C) to array probes overnight. The chips were washed, stained, and scanned to generate a CEL file containing the fluorescence intensities associated with each probe. Hybridization scores for each probe set were calculated from the fluorescence intensities of individual probes and then processed to give abundance data for the associated OTUs. PhyloChip microarray files containing raw hybridization intensity scores have been deposited on the Greengenes server^1^.

Hybridization scores for each chip were scaled to an internal standard, then log_2_-transformed. The detailed criteria for assigning probe hybridization scores were as previously described (Brodie et al., 2007; Hazen et al., 2010). OTU and higher-level taxa selection from raw G2 PhyloChip data followed Hazen et al. (2010). For presence/absence assessment of PhyloChip arrays, PhyCA parameters were selected to mimic the pf ≥ 0.9 criterion (pf, positive fraction of perfect probe matches for an OTU) that has been formerly used in CEL analysis. In the first stage of analysis for each OTU probe set, probe pair hybridization scores, denoted “*r*,” were ranked and probe set quartile boundaries (rQ1, rQ2, and rQ3) were determined. For a probe set to pass this stage of analysis, we required rQ1 ≥ 0.379, rQ2 ≥ 0.565, rQ3 ≥ 0.82, and pf ≥ 0.93. Passing OTUs were then evaluated for cross-hybridization potential in a second stage of analysis, using a cutoff point of rxQ3 ≥ 0.515 (where rxQ3 denotes the third quartile boundary of ranked, cross-hybridization adjusted scores). OTUs passing both stages were called present. All OTUs present in at least one sample were included in calculation of OTU rank abundances for each sample.

For mats SEEP 1, 3, 4, 6–9, 11, and 13, aliquots of amplified 16S rRNA gene PCR product described above were cloned using a PCR Cloning Kit (Qiagen). Plasmids were purified and sequenced by the U.C. Berkeley DNA Sequencing Facility. Clone library sequences were assessed for quality and assembled using Geneious (v.5.5.6; Biomatters Ltd.). Following assembly, sequences were screened for putative chimeras using the Mothur implementation of Chimera Slayer (Schloss et al., 2009), Mallard (Ashelford et al., 2006), and BLAST searches (Altschul et al., 1990) of all sequences with suspected chimeras. Sequences that potentially represented chimeras were discarded before further clone library analysis.

### SEEP Fatty Acid and DNA Correlation Analysis

To identify OTUs that may assimilate ^13^C-depleted methane or ^13^C-enriched CO_2_ to characteristic lipids, we looked for correlations between the PhyloChip-based taxon abundance and the fatty acid δ^13^C values of SEEP samples. We limited this analysis to taxa that ranked within the top 25 OTUs from one or more SEEP samples, and to the eight fatty acids that were detected in at least three SEEP samples and that showed ≥10‰ variation in δ^13^C values across SEEP samples. We tested for association between each fatty acid’s measured δ^13^C values and each OTU’s rank abundances using Spearman’s rho, applying the Benjamini and Hochberg false discovery rate correction to the resulting *p*-values to control for multiple comparisons to each OTU (Benjamini and Hochberg, 1995). We applied a significance threshold of 0.05 to the FDR-corrected *p*-values.

### SIP Sampling and Incubations

In June 2008, an additional *in situ* colonization device was deployed at Shane Seep to support the growth of mats for use in laboratory SIP enrichments. After 6–12 weeks of undisturbed growth *in situ*, individual mat samples were recovered by SCUBA divers. Harvested mats were collected in sealed containers, with seawater from Shane Seep, and immediately stored near *in situ* temperature (12°C). Incubations were begun on the same day. Incubations lasted 111 (DNA-SIP) to 171 (Lipid-SIP) hours based on headspace gas consumption, and were conducted in the dark, at *in situ* temperature, in re-sealable Tedlar septum bags. Mat samples were immersed in 250 mL of seawater from Shane Seep under a 170 mL headspace [^13^CH_4_ (^13^C isotopic purity, 99.9%), 3.9–4.9%; O_2_, 18.8–19.5%; N_2_, 74.6–77.2%; CO_2_, 0.1–1%] at atmospheric pressure. Methane consumption and carbon dioxide production were monitored throughout the incubations by thermal conductivity detector gas chromatography (GC-TCD), using a 3000A MicroGC (Agilent).

### SIP Lipids Extraction and Analysis

Total lipid extracts (TLEs) were obtained from the mat biomass using a modified Bligh and Dyer protocol (Sturt et al., 2004), after adding an internal standard (phosphatidylcholine C_21:0/21:0_) and 3 g of combusted sea sand. The obtained TLEs were stored at −20°C and analysis of IPLs was performed on a Thermo Finnigan Surveyor high-performance liquid chromatography system coupled to a Thermo Finnigan LCQ DecaXP Plus ion-trap mass spectrometer via electrospray interface (HPLC-ESI-ITMS^n^) under conditions described previously (Sturt et al., 2004). Compound identification was achieved by monitoring exact masses of possible parent ions (present mainly as H^+^ and NH_4_^+^ adducts) in combination with characteristic fragmentation patterns (Sturt et al., 2004). The reported relative distribution of microbial lipids is based on the peak areas of the respective molecular ions without differentiating for potential differences in response factors; the data should therefore be viewed as semi-quantitative.

For the Lipid-SIP experiments, an aliquot of the TLE was saponified with 6% KOH to release the polar lipid-derived fatty acids (PLFAs; Kellermann et al., 2012). Subsequently, the neutral lipids were removed with n-hexane from the basic solution, and the FAs extracted with n-hexane after acidifying the remaining solution to a pH close to 1, by adding dropwise concentrated HCl. PLFAs were subjected to GC-mass spectrometry as methyl esters (FAMEs) using 14% BF_3_ in MeOH.

Fatty acid methyl esters were identified and quantified by gas chromatography–mass spectrometry (GC–MS) system as described by Kellermann et al. (2012). The carbon isotopic composition was determined using GC-isotope ratio-MS (GC-irMS) at least in duplicate measurements (Trace GC Ultra coupled to a GC-IsoLink/ConFlow IV interface and a Delta V Plus irMS; all from Thermo Scientific). Compounds were oxidized in a combustion reactor at 940°C (Thermo Finnigan Combustion Interface-II). The stable carbon isotope values are expressed in the δ-notation in per mill (‰) as deviation of the isotope ratio from the reference standards. The analytical error was <0.5‰ for non-labeled δ^13^C values. δ^13^C values were corrected for additional carbon introduced during derivatization.

### SIP DNA Extraction and Separation

Nucleic acids were extracted from one sample at *t* = 0 to provide a control of non-^13^C-labeled DNA. At *t* = 111 h, one ^13^CH_4_ incubation was sacrificed for endpoint DNA. Nucleic acids were purified from *t* = 0 and ^13^CH_4_ samples as described above (see SEEP DNA analysis). For the ^13^CH_4_ (“SIP”) and *t* = 0 (initial time point, “INIT”) samples, we separated DNA by density via ultracentrifugation in a solution of cesium chloride (Neufeld et al., 2007), recovering twelve fractions from each sample’s density gradient. DNA was quantified in density fractions using the Bioanalyzer 2100 and high-sensitivity dsDNA kit (Agilent Biosciences). In both the SIP and INIT density gradients, seven fractions (SIP1–7, 1.697–1.763 g/mL; and INIT1–7, 1.597–1.753 g/mL) contained sufficient DNA to permit further analysis.

### SIP DNA Analyses

DNA from 14 samples, SIP1–7 and INIT1–7, was subjected to 16S rRNA gene amplification using primers 27F and 1492R, as above. We performed PhyloChip analysis on gradient fractions DNA-SIP1–7 and INIT1–7. PhyloChip sample processing and probe hybridization scoring were as described above. To examine taxonomic associations for organisms that may assimilate seep-derived carbon to biomass, we focused our analysis on the subfamilies containing OTUs whose relative abundance was significantly correlated to fatty acid δ^13^C values in SEEP samples. From this list of subfamilies, we chose OTUs that ranked among the top 50 taxa in at least one SIP or INIT gradient fraction. We then compared taxon abundance shifts across each gradient, using heatmaps to reveal any density-driven patterns. We cloned and sequenced amplicons (as described above) from the DNA-SIP2 (1.707 g/mL) and DNA-SIP4 (1.731 g/mL) fractions and in addition to classifying all clone library sequences against the RDP database, we used ClustalW (Larkin et al., 2007) to assess pairwise alignments with GenBank sequences corresponding to PhyloChip OTUs for ^13^C-enriched methanotroph taxa. Sequences from the SIP 16S rDNA clone libraries were submitted to the GenBank database under the accession numbers JX567952–JX568074.

## Results and Discussion

### Initial Observations and Community Composition

To examine the compositional variability and methanotrophic potential of seep mats, we obtained samples from artificial growth surfaces (**Figure 1A**) tethered 20–50 cm above active gas vents at Shane Seep. *In situ*, mats rapidly developed on the growth device deployed within the seep field, but no mat growth was observed on the device deployed 30 m outside of the seep. We observed strong variations in the color and texture of individual mats, suggesting local spatial heterogeneity in bacterial community composition (**Figure 1B**). Mat morphologies ranged from thin, sheet-like biofilms to filamentous structures, and colors varied from green and brown to white. These differences occur within individual mats and between different mat samples. Our initial observations suggested that morphologically diverse microbes can become prevalent in seep mats, leading us to ask whether these organisms also exploit diverse metabolic strategies.

To gain a first look at that diversity, we analyzed the bacterial 16S rRNA gene by PhyloChip. We detected a total of 1878 bacterial OTUs from eight seep mat samples, averaging 817 ± 320 detected OTUs per mat. Based on OTU incidence in these eight samples, we calculated Chao richness of ∼2346 OTUs (SE 52.9) for the seep mat community. It should be noted that this is an estimate of richness at the subfamily level, the limit of PhyloChip resolution; the species-level richness is expected to be markedly greater. Notably, the number of distinct subfamilies predicted in these mats is roughly comparable with the number of species in previously studied mat communities, including mats believed to be among the most diverse marine ecosystems known (Bolhuis and Stal, 2011; Mobberley et al., 2012; Song et al., 2013). Shane Seep mats may therefore represent a noteworthy community for bacterial diversity in a marine ecosystem.

**FIGURE 2:**
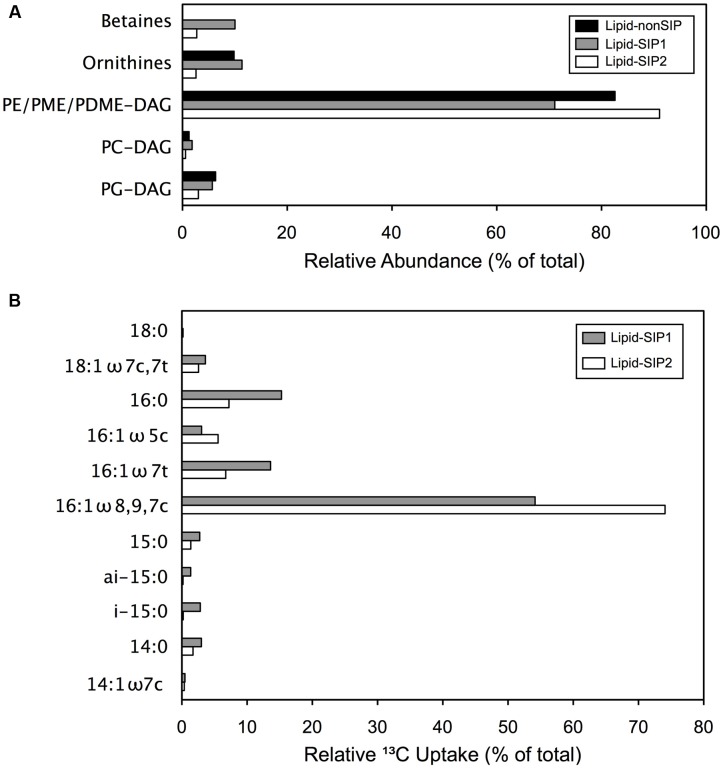
**Comparative IPL distribution and FA ^13^C enrichment: (A)** Relative IPL (%) abundances in methane enrichment samples. **(B)** Proportion of ^13^C assimilated to specific fatty acids, determined from relative (%) abundances and δ^13^C values for each lipid.

To further examine the bacterial composition of these mats, we assessed the variability of IPL across samples. Since the polar head groups of these macromolecules are prone to degradation shortly after cell death, IPLs provide a useful proxy for live cell biomass in microbial samples (Rütters et al., 2002; Lipp and Hinrichs, 2009; Logemann et al., 2011). Mat biomass from COP samples comprised up to 271 μg/g IPL, exceeding previously measured concentrations for surface sediments from other marine environments (Schubotz et al., 2009, 2011). Diacylglycerol lipids (DAG) with head groups of phosphatidylethanolamine (PE-), phosphatidyl-(N)-methylethanolamine (PME-), or phosphatidyl-(N,N)-dimethylethanolamine (PDME-), contributed between 71 and 92% of the total IPL pool (**Figure 2A**). These findings suggest that the mats are predominantly composed of bacterial cells, but may include a substantial population of eukaryotes, as discussed in detail below.

### Microbial Mats Harbor Active Methanotrophs

We next asked whether the mat ecosystem could constitute a significant biofilter on methane in shallow seeps, and which taxa were associated with methane carbon uptake. We deployed an additional benthic growth device at Shane Seep to obtain intact mats for SIP with ^13^CH_4_. We retrieved five intact microbial mats. One was immediately frozen upon retrieval to provide a baseline (“INIT” sample), one was incubated with unlabeled CH_4_, and three were incubated with ^13^CH_4_. Monitoring of headspace gasses indicated CH_4_ and O_2_ consumption and CO_2_ production prior to harvesting and sample processing (Supplementary Figure S2). Over the course of the 4–7 days incubations, individual mats (100 cm^2^) consumed 0.1–0.4 mmol CH_4_. If methane oxidation *in situ* were to occur at a comparable rate, then a carpet of active methanotrophic mats covering the entire ∼1350 m^2^ seep area could consume just 7.7 mol CH_4_ day^-1^, ∼0.006% of the 122,900 mol of methane released daily from this location (Washburn et al., 2005; Kinnaman et al., 2007). This low value reflects the partitioning of seep gas between ebullition and dissolution at this shallow seep: at just ∼20 m water depth, the ambient pressure of ∼3 atm permits the large majority of seeped methane to escape dissolution en route to the atmosphere, leaving a minor fraction available for consumption by the mats. Nonetheless, the observation that mats form readily on benthic growth devices deployed within the seep but fail to form on a device outside the seep suggests that seep gas might play a key role in stimulating microbial growth within the shallow seep ecosystem.

We extracted lipids from two ^13^CH_4_ incubations (“Lipid-SIP” samples) and the unlabeled CH_4_ control incubation (“Lipid-nonSIP”). To assess the role of methanotrophic activity in microbial mat development, we measured the δ^13^C signatures of specific fatty acids in Lipid-SIP and Lipid-nonSIP samples (Supplementary Table S1), as well as the relative abundance of individual lipids in the total extracted pool. Monounsaturated 16-carbon and 18-carbon fatty acids dominated each sample, comprising more than 70% of fatty acids in all samples. The highest degree of ^13^C enrichment in Lipid-SIP samples was observed in co-eluted 16:1-ω7c, 16:1-ω8, and 16:1-ω9 FAs (**Figure 2B**), consistent with ^13^C depletion of the same 16:1 FAs in the Lipid-nonSIP sample. SIP also revealed weaker ^13^C enrichment of 15:0, 16:0, 16:1-ω5, 16:1-ω7t, and 18:0 FAs, though these relatively low-abundance FAs contribute comparatively little ^13^C to mat biomass. By contrast, the three 16:1 isomers showing the most substantial ^13^C enrichment also constituted the largest fraction of the FA pool, accounting for the bulk of ^13^C incorporation. While the -ω8 and -ω9 isomers of 16:1 FAs are minor lipids for most bacterial phyla, they are the dominant membrane component in known aerobic methanotrophs belonging to the family Methylococcaceae (Bowman, 2006; Tavormina et al., 2015, 2017). These findings clearly demonstrate that the mat ecosystem harbors active methanotrophs.

To establish taxonomic affiliations for primary methane consumers in the microbial mats, we harvested DNA from the remaining ^13^CH_4_ SIP incubation (“DNA-SIP” sample, **Figure 1C**) for comparison to DNA from the INIT sample. Following density fractionation, seven initial-time fractions (INIT1–7) and seven ^13^C fractions (DNA-SIP1–7) contained sufficient DNA for further analysis. As a first look at density-driven taxon abundance shifts, we built clone libraries from two DNA-SIP fractions, DNA-SIP2 (1.707 g/mL) and DNA-SIP4 (1.731 g/mL). Six families accounted for >95% of the DNA-SIP2 sequences, with Rhodobacteraceae the most abundant at 35.6% and Methylococcaceae at 10.2% (Supplementary Figure S3). While the same families dominated the DNA-SIP4 library, the relative abundances differed, with just 11% of sequences from Rhodobacteraceae, but 26.1% from Methylococcaceae (Supplementary Figure S3). The non-methanotrophic and non-methylotrophic families Flavobacteriaceae and Campylobacteraceae were also substantially more abundant in DNA-SIP4 than in DNA-SIP2, highlighting the need for a deeper examination of community structure along the full SIP and INIT gradients to determine which differences were driven by ^13^C enrichment.

**FIGURE 3:**
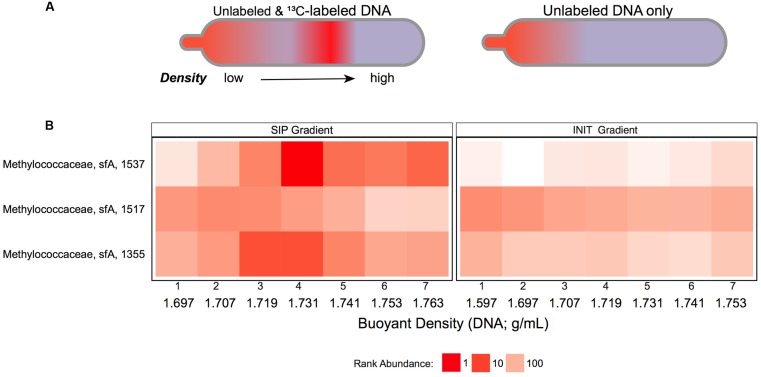
**Methanotroph abundance variability across DNA density gradients: (A)** Schematic of theoretical density gradients of total community DNA in centrifuge tubes from SIP-labeling (left) and nonSIP (right) experiments. **(B)** Heatmap of ranked abundances from OTUs (OTU # indicated on *Y*-axis), belonging to Methylococcaceae subfamily-A in the DNA-SIP (left) and INIT (right) density gradients. Fractions 1–7 from each gradient are labeled with their buoyant densities (g/mL).

Accordingly, we used PhyloChip microarrays to assess fractions DNA-SIP1–7 and INIT1–7. Two Methylococcaceae taxa, OTUs 1537 and 1355, were detected at comparable abundances in the lightest fractions of the DNA-SIP and INIT gradients, but reached much higher abundance in denser SIP fractions (1.731–1.763 g/mL) than in the corresponding INIT fractions (**Figure 3**). This pattern suggests that Methylococcaceae OTUs 1537 and 1355—both known methanotrophs—became ^13^C-enriched in our DNA-SIP incubation. A third Methylococcaceae taxon, OTU 1517, was present in both DNA-SIP and INIT gradients but did not show ^13^C-dependent density shifts. More dense fractions were enriched for Sulfurovum (Supplementary Figure S4) and unclassified Sulfurovumales (Supplementary Figure S5) in SIP gradients as compared with INIT gradients. While Methylophaga (Supplementary Figure S6) and other unclassified bacteria (Supplementary Figure S5) echoed this pattern to some extent, the effect was not substantial.

### Evidence for *In situ* CH_4_ and CO_2_ Assimilation

Stable isotope probing-based examination of the mats identified the taxa incorporating methane carbon under closed-system conditions. However, this signal may derive in part from cross-feeding; e.g., the shift of sulfur-oxidizing taxa to denser fractions in incubations with ^13^CH_4_ likely reflects fixation of ^13^CO_2_ released by methanotrophs. To address this challenge, we made use of the natural ^13^C depletion of seep methane (δ^13^C = -50.5 to -53.9) and the natural ^13^C enrichment of seep CO_2_ (δ^13^C = 15.8 to 18.4) to investigate relationships between OTU abundances and lipid isotope signatures in the SEEP samples.

We analyzed the fatty acid composition of the 14 SEEP mat samples (**Figure 1C**), detecting a total of 25 unique fatty acids. Of these, 12 FAs were abundant, each constituting ≥5% of the lipids in at least one SEEP sample (Supplementary Table S2). Consistent with our Lipid-SIP results and with previous work at Shane Seep (Ding and Valentine, 2008), we detected 16:1 fatty acids at high relative abundance. Strikingly, not only did total mat biomass δ^13^C vary by 26‰ (Supplementary Table S3), the δ^13^C values for the major FAs varied by as much as 39 ‰ across the 14 SEEP samples (Supplementary Table S4). Because each FA is typically produced by a range of taxa, the aggregate δ^13^C for a given FA will be a weighted average of the FA δ^13^C in the contributing taxa, with a given clade influencing the aggregate signal in proportion to its level of FA production and abundance in the community.

**FIGURE 4:**
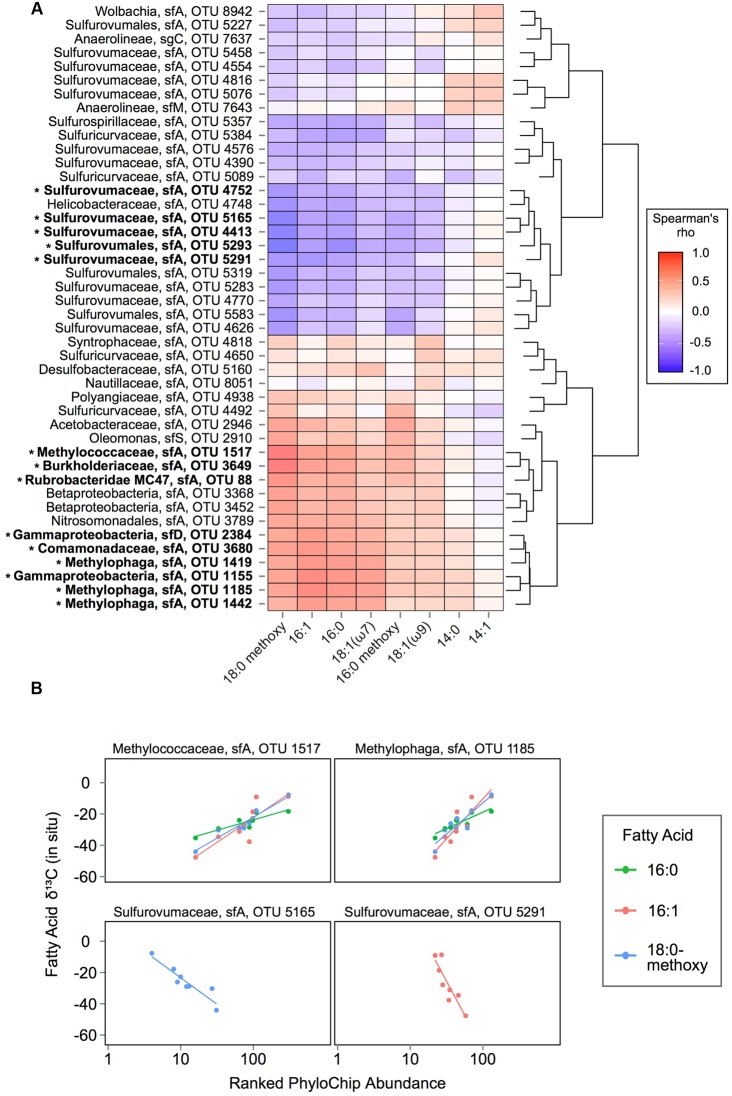
**Correlation analysis of fatty acid ^13^C signatures vs. taxa abundances: (A)** Spearman’s rho analysis of ranked OTU abundances versus fatty acid δ^13^C values in SEEP samples. OTUs are ordered by hierarchical clustering on Euclidean distances from Spearman’s rho values. Taxonomic affiliations (lineage, subfamily, and PhyloChip OTU number) are displayed at left for each row. OTUs that have a significant association with at least one fatty acid’s δ^13^C values are highlighted in bold and with an asterisk mark. **(B)** Selected examples of OTUs with ranked abundances significantly (*p* ≤ 0.05) associated with fatty acid δ^13^C values in SEEP samples.

Using data from the 8 SEEP mats for which both lipids and DNA were analyzed, we therefore asked whether the observed FA isotope signature variations occurred in tandem with variation in the abundance of key taxa as detected by PhyloChip, to determine whether specific populations might be linked to the assimilation of either ^13^C-enriched or ^13^C-depleted seep gasses (**Figure 4**). We considered only those OTUs that ranked in the top 25 in at least one SEEP sample, and the 8 FAs for which (a) complete or nearly complete data was available and (b) the range of δ^13^C variation was greater than 10‰. We found significant correlations (with false discovery rate for adjusted *p*-values at 0.05) between OTU abundance and the δ^13^C signatures of four FAs: 16:1, 16:0, 18:0 methoxy and 18:1-ω7. For 16:1, 16:0, and 18:0 methoxy FAs, ^13^C-depletion was positively correlated with the ranked abundance of 8–9 OTUs representing Burkholderiaceae, Comamonadaceae, Methylococcaceae, Methylophaga (2–3 OTUs), Rubrobacteridae, and unclassified -proteobacteria (2 OTUs). ^13^C-depletion of 18:1-ω7 FAs was positively correlated with a subset of these taxa: one unclassified -proteobacteria and three Methylophaga OTUs. By contrast, ^13^C-depletion of 16:1 and 18:0 methoxy FAs was negatively correlated with the ranked abundance of up to 4 OTUs representing Sulfurovumaceae and one OTU from unclassified Sulfurovumales. Ranked abundance of the latter OTU was also negatively correlated with ^13^C-depletion of 16:0 FAs.

The correlation we observed in SEEP samples between the abundance of Methylococcaceae and the ^13^C-depletion of 16:1 and 16:0 fatty acids offers corroborating evidence that Methylococcaceae actively convert ^13^C-depleted seep methane to biomass in microbial mats at shallow, cold marine hydrocarbon seeps. Importantly, these findings also point to substantial uptake of methane-derived carbon by mat organisms downstream of the primary methanotrophs. Importantly, the strength of each relationship observed between ^13^C-depletion/enrichment and OTU abundance (i.e., Spearman’s rho and slope) might reflect the extent to which isotopically intermediate carbon was incorporated for a particular fatty acid. The present study offers clear evidence of Methylophaga activity in Shane Seep mats, with members of these methylotrophic taxa occurring at high relative abundances in SEEP mats with ^13^C-depleted lipids. The Methylophaga OTUs we detected have not been shown to oxidize methane, instead relying on methanol as a carbon source (Lidstrom, 2006). Their abundance and association with ^13^C-depleted methane carbon from Shane Seep suggests that Methylophaga are key players in cross-feeding on partially oxidized methane metabolites within these mats, similar to the pelagic bacterial communities that responded to the Deepwater Horizon blowout (Kessler et al., 2011; Redmond and Valentine, 2012).

**FIGURE 5:**
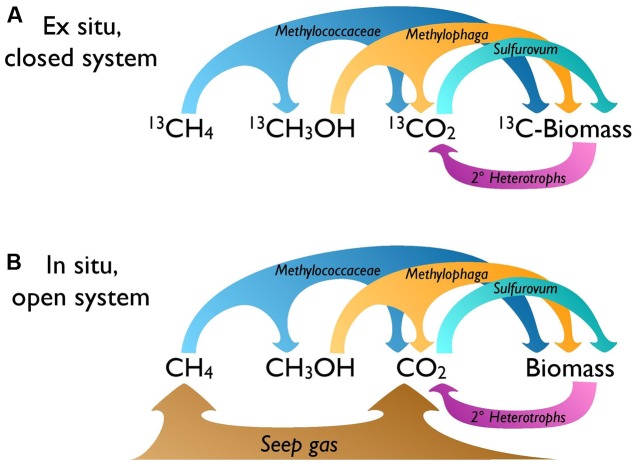
**Microbial food web for the seep mat community.** Bacterial carbon transfer under **(A)** *ex situ* SIP and **(B)** *in situ* conditions. *Ex situ*, the CO_2_ available to autotrophs like Sulfurovum is the product of methane oxidation; *in situ*, more CO_2_ is expected to be supplied directly by the gas seep.

Farther downstream, fully oxidized methane carbon appears to fuel autotrophy by sulfide oxidizers under closed-system SIP conditions, leading to ^13^C enrichment of Sulfurovumales in the DNA-SIP gradient (**Figure 5A**). *In situ*, where CO_2_ constitutes up to 17% of dissolved gasses at Shane Seep (Kinnaman et al., 2007), the enriched isotopic fingerprint of lipids linked to autotrophic sulfide-oxidizers suggests preferential use of ^13^C-enriched CO_2_ from seep gas over ^13^C-depleted CO_2_ from upstream methanotrophy or methylotrophy (**Figure 5B**). Bacteria capable of autotrophic sulfide oxidation were consistently among the most abundant groups detected by PhyloChip analysis of SEEP samples, with Sulfuricurvum, Sulfurospirillum, and Sulfurovum (respectively, PhyloChip Sulfuricurvaceae, Sulfurospirillaceae, and Sulfurovumaceae OTUs) among the top 10 abundant taxa in all eight mats analyzed (Supplementary Table S5).

To examine the mats for cellular sulfur inclusions, a common feature of sulfide-oxidizing bacteria, we applied scanning electron microscopy (SEM) and energy-dispersive X-ray spectrometry (EDS). Apparently filamentous cell assemblages were observed that contained putative granules (Supplementary Figures S7A–C). EDS analysis revealed that the granule-like structures are almost entirely composed of sulfur (Supplementary Figure S7D). This evidence suggests that the abundant filamentous cells can store sulfur in cellular inclusions, which in turn might be a characteristic of sulfide-oxidizing phyla that dominate the shallow seep mat ecosystem. Sulfide-oxidizing bacteria are often the most abundant organisms found in benthic mats in both methane seep and non-seep environments, where reduced sulfur is transported upward from the subsurface (Nelson et al., 1989; Zhang C.L. et al., 2005; Moussard et al., 2006; Gilhooly et al., 2007; Valentine et al., 2016).

### Abundant Mat Phyla in Auxiliary Niches

While seep gas appears to support methanotrophs, methylotrophs, and sulfur-oxidizers in the mats, these taxa represent a small fraction of mat biodiversity. Because the Coal Oil Point seeps release oil and tar as well as gas (Allen et al., 1970; Wardlaw et al., 2008; Farwell et al., 2009), we next asked whether our PhyloChip assays identified any taxa whose cultured relatives exhibit a capacity for degradation of petroleum compounds. Out of the top 50 ranked OTUs across all SEEP samples, we found three taxa affiliated with oil-degrading representatives, including members of the Alcanivoraceae family, the genus Petrobacter, and the genus Oleomonas (Supplementary Table S5). Notably, one Oleomonas OTU was detected among the top 10 ranked taxa in four SEEP samples. Cultured organisms belonging to this lineage have exhibited favorable growth on ethanol, propanol, and butanol, and are also capable of oxidizing aliphatic and aromatic hydrocarbons (Kanamori et al., 2002). Though future work is needed to determine the role of these taxa in the COP mats, we hypothesize that oil-degrading bacteria might contribute to the preferential growth of microbial mats within the seep field.

Looking beyond hydrocarbon consumption, one myxobacterial OTU (Polyangiaceae OTU 4938), most closely related to *Sorangium cellulosum*, was identified amongst the top five most abundant taxa in samples SEEP7, SEEP9, and SEEP13 (Supplementary Table S5). In marine sediments these organisms are found as individual cells, although they can also form macroscopic fruiting structures that may resemble a developing biofilm on the seafloor (Zhang Y.Q. et al., 2005; Brinkhoff et al., 2012). Given that cultured relatives are typified by gliding motility, swarming, and biofilm development, the repeated observation of Myxobacteria at high abundance in the mats raises questions about a potential role as early colonizers in driving mat formation at shallow seeps. The occurrence of dominant myxobacteria in COP mats sets these mats apart from previously examined COP sediments (Redmond et al., 2010), and likewise from Beggiatoa-dominated mats from deep marine seeps (Niemann et al., 2006; Lösekann et al., 2007).

While the seep mats appear to be predominantly bacterial based on IPL composition, the IPL distributions also pointed to a potential biomass contribution from eukaryotes. In particular, 10.0% of Lipid-SIP1 IPLs and 2.7% of Lipid-SIP2 IPLs were betaine lipids (**Figure 2A**). Although the source of betaine lipids in these mats is unknown, betaine lipid abundance in various marine samples has been linked to eukaryotic phytoplankton (Kato et al., 1996; Popendorf et al., 2011), and previous work has measured high chlorophyll concentrations in COP microbial mats (Montagna and Spies, 1985). While phytoplankton are known to substitute phospholipids with aminolipids in oligotrophic marine environments (Van Mooy et al., 2009), it is unlikely that the COP nearshore ecosystem experiences P-limitation. To investigate whether the COP microbial mats harbor eukaryotic algae, we cloned and sequenced the 18S rRNA gene from SEEP3 (Supplementary Table S6). The sequence library was predominantly affiliated with Thalassiosirales (diatoms; 28 clones) and Gymnodiniales (dinoflagellates; 26 clones), and remaining sequences appear linked to a broad range of additional taxa. Taken together with the betaine lipids captured by our IPL analysis, these findings suggest a role for phototrophy and photomixotrophy in Shane Seep mats, perhaps supplying oxygen to aerobic processes within these microbial mats.

## Conclusion

In this study, we used parallel lipid and DNA analysis to explore the microbial mats that line the seafloor at Shane Seep, part of one of the world’s most vigorous methane seep systems. Despite substantial variability in benthic microbial mats in the fluctuating environment of the upper continental shelf, lipid profiling of a set of natural mat samples showed clear evidence for carbon incorporation by methanotrophs. SIP *ex situ* enabled the identification of key methanotrophic bacteria; our correlation of *in situ* taxon abundances with natural ^13^C of biomass additionally points to activity by methylotrophic and sulfide-oxidizing bacteria that acquire carbon from partially oxidized methane and carbon dioxide, respectively. Their coexistence *in situ* opens a route for sulfide oxidizers to use direct trophic interactions with methanotrophic and/or methylotrophic neighbors to supplement the CO_2_ they capture directly from seep gas. Alongside these niches, the seep’s location in the euphotic zone appears to support the growth of photosynthetic eukaryotes. Although the mats act as a sink for only a small fraction of seep gas, the three distinct pathways of biomass productivity available in this shallow seep support one of the most diverse marine microbial assemblages yet studied.

## Author Contributions

BP, HD, and DV designed the experiments and conducted sampling for the study. HD and MK carried out lipid measurements and BP, HD, MK, SB, and DV performed lipid data analyses. BP, MR, SB, GA, and DV conducted molecular DNA experiments and data analysis. BP and DV wrote the manuscript.

## Conflict of Interest Statement

The authors declare that the research was conducted in the absence of any commercial or financial relationships that could be construed as a potential conflict of interest.

## Footnotes

1 http://greengenes.lbl.gov/Download/Microarray_Data/Seep-mats_Paul_2014/
