# Supplementary material for: Methane-Oxidizing Bacteria Shunt Carbon to Microbial Mats at a Marine Hydrocarbon Seep
Source: Front Microbiol. 2017 Feb 27;8:186. doi: 10.3389/fmicb.2017.00186 (PMC5326789; doi:10.3389/fmicb.2017.00186)
Supplement: Supplementary file 1 [file Data_Sheet_1.PDF]

Supplemental Tables and Figures

Table S1. Relative abundances by mass and $\delta^{13}C$ values for fatty acid (FA) types detected in Lipid-SIP1, Lipid-SIP2, and Lipid-nonSIP samples.

Fatty Acid (FA)	Relative Abundance (% FA)			$\delta^{13}C$ (‰)		
	Lipid-nonSIP	Lipid-SIP1	Lipid-SIP2	Lipid-nonSIP	Lipid-SIP1	Lipid-SIP2
14:1(ω7c)	0.7	1.6	5.3	n.d.	64	22
14:0	2.8	4.4	5.8	-32	130	84
i-15:0	1.9	2.3	0.8	-35	259	68
ai-15:0	0.8	1.0	0.2	n.d.	281	119
15:0	1.9	2.3	0.6	-43	233	786
16:1(ω8,9,7c)	39.4	46.2	49.9	-44	232	536
16:1(ω7t)	15.0	17.6	9.2	-50	133	234
C16:1(ω5c)	6.1	3.9	6.5	-54	130	280
C16:0	18.6	14.5	9.0	-42	208	272
C18:1(ω7c,7t)	10.0	5.6	12.3	-35	119	47
18:0	2.9	0.7	0.4	n.d.	n.d.	134

n.d., not detected

Table S2. Relative abundances by mass of the 12 major fatty acid (FA) types detected in SEEP1–14. Major FAs are those that exceeded 5% abundance in at least one sample.

	Percent of Fatty Acid Pool											
Sample	12:0	14:1	14:0	15:0	16:1	16:1(5)	16:0	16:0 9-methoxy	18:1(9)	18:1(7)	18:0	18:0 methoxy	Other FAs
SEEP1	2.0	3.2	11.8	4.1	23.9	0.9	23.7	1.2	4.7	4.5	4.5	0.2	15.4
SEEP2	2.9	1.3	17.6	6.5	8.2	0.6	31.0	4.5	2.5	1.1	7.1	2.1	14.6
SEEP3	2.9	2.9	16.6	4.8	18.1	0.7	27.4	0.3	5.6	3.3	3.9	1.2	12.2
SEEP4	1.3	n.m.	15.0	6.0	2.5	n.d.	33.8	11.0	0.1	1.0	9.0	7.5	12.8
SEEP5	3.7	4.6	15.1	4.3	15.9	n.d.	21.9	8.0	1.8	2.4	5.9	1.0	15.5
SEEP6	2.0	4.4	11.4	3.7	32.9	n.m.	21.1	1.1	1.0	2.8	3.6	0.8	15.2
SEEP7	10.9	7.2	16.1	0.6	15.6	n.d.	4.1	9.7	3.2	12.6	9.8	3.2	7.2
SEEP8	9.4	8.1	17.3	1.3	22.2	n.m.	6.9	8.8	4.2	4.8	5.3	7.0	4.6
SEEP9	1.9	4.2	7.0	1.5	46.7	2.8	12.7	1.5	2.1	11.9	1.3	0.4	6.1
SEEP10	9.0	4.7	7.3	0.6	14.8	n.d.	6.5	27.5	2.1	9.8	2.7	5.9	9.1
SEEP11	1.0	3.7	4.6	0.5	14.3	1.0	58.4	2.2	1.0	9.5	0.9	0.9	1.9
SEEP12	1.2	2.1	5.3	1.7	44.3	6.2	14.1	3.1	1.2	11.8	1.5	1.4	5.9
SEEP13	4.4	10.7	11.4	1.2	42.9	4.4	10.7	0.6	0.8	6.6	0.6	0.9	5.0
SEEP14	7.4	15.1	14.1	1.2	37.2	n.d.	12.0	2.8	0.3	3.5	0.6	1.0	4.8

n.m., concentration not measured
n.d., not detected

Table S3. Elemental composition (as percent of total dry mass) and $\delta^{13}C$ values for environmental samples SEEP1–14.

Sample	% S	% H	% N	% C	$\delta^{13}C$
SEEP1	14.3	7.3	12.1	66.3	-11.7
SEEP2	14.7	9.2	12.3	63.8	-20.6
SEEP3	9.3	9.9	11.5	69.3	-37.5
SEEP4	4.4	10.6	12.6	72.4	-27.9
SEEP5	5.4	8.4	13.9	72.3	-16.7
SEEP6	17.9	8.2	11.3	62.6	-11.3
SEEP7	4.4	8.8	14.7	72.1	-20.4
SEEP8	9.9	10.4	11.4	68.3	-23.8
SEEP9	5.1	10.6	13.9	70.5	-31.1
SEEP10	3.5	10.4	14.9	71.3	-33.2
SEEP11	11.7	8.4	13.9	65.9	-17.1
SEEP12	6.8	9.8	13.8	69.7	-24.7
SEEP13	11.2	9.4	13.7	65.7	-34.3
SEEP14	13.1	8.2	14.1	64.5	-19.9

Table S4. δ^{13}C values for the 12 major fatty acid (FA) types detected in SEEP1–14. Major FAs are those that exceeded 5% abundance in at least one sample.

	δ¹³C (‰)											
Sample	12:0	14:1	14:0	15:0	16:1	16:1(5)	16:0	16:0 9-methoxy	18:1(9)	18:1(7)	18:0	18:0 methoxy
SEEP1	-29	-11	-22	-28	-9	n.m.	-19	-21	-12	-8	-23	-18
SEEP2	-32	-25	-28	-28	-26	n.m.	-25	-21	-27	-20	-25	-22
SEEP3	-30	-29	-29	-29	-38	-49	-29	n.m.	-27	-35	-27	-26
SEEP4	-31	-37	-26	-29	-19	n.d.	-24	-18	-32	-22	-23	-23
SEEP5	-26	-20	-22	-27	-26	n.d.	-23	-22	-25	-6	-27	-17
SEEP6	-25	-9	-21	-28	-28	-40	-27	-48	-27	-13	-27	-29
SEEP7	-28	-8	-20	-30	-31	n.d.	-24	-32	-20	-12	-21	-29
SEEP8	-27	-4	-19	-28	-25	-34	-25	-22	-36	-23	-21	-21
SEEP9	-28	-1	-16	-31	-35	n.m.	-29	-31	-24	-26	-25	-30
SEEP10	-25	-5	-18	-30	-36	n.d.	-28	-32	-23	-20	-23	-19
SEEP11	-30	-2	-10	-30	-9	n.m.	-18	-12	-4	-8	-21	-8
SEEP12	-28	-2	-14	-32	-35	n.m.	-20	-27	-18	-20	-22	-27
SEEP13	-29	-13	-29	-32	-48	n.m.	-35	-41	-30	-34	-21	-44
SEEP14	-28	-3	-14	-31	-31	n.d.	-26	-24	-20	-22	-19	-30

n.m., fractionation not measured
n.d., not detected

Table S5. Taxa dominating environmental PhyloChip samples. Taxa ranked among the top 50 OTUs in one or more SEEP samples are shown. For top ranking OTUs within each taxonomic classification, the most and least abundant ranks and corresponding SEEP sample are shown.

Taxon:	Top Rank / Sample	Lowest Rank / Sample
Acetobacteraceae	14th / SEEP7	136th / SEEP11
Alcanivoracaceae	35th / SEEP9	225th / SEEP11
Alteromonas	41st / SEEP7	558th / SEEP11
Anaerolineae	10th / SEEP3	101st / SEEP11
Burkholderiaceae	8th / SEEP13	167th / SEEP11
Campylobacteraceae	32nd / SEEP11	158th / SEEP9
Comamonadaceae	12th / SEEP13	229th / SEEP11
Cytophaga	41st / SEEP4	165th / SEEP6
Desulfobacteraceae	12th / SEEP3	25 / SEEP11
Enterobacteriales	50th / SEEP9	384th / SEEP11
Helicobacteraceae	16th / SEEP11	48th / SEEP13
Hyphomonadaceae	48th / SEEP7	205th / SEEP11
Marinobacter	42nd / SEEP13	142nd / SEEP6
Methylobacteriaceae	36th / SEEP13	903rd / SEEP11
Methylococcaceae	16th / SEEP13	304th / SEEP11
Methylophaga	3rd / SEEP13	62nd / SEEP11
Nautillaceae	9th / SEEP4	32nd / SEEP13
Oleomonas	5th / SEEP9	139th / SEEP11
Petrobacter	35th / SEEP13	667th / SEEP11
Polyangiaceae	3rd / SEEP7	21st / SEEP11
Pseudomonadaceae	38th / SEEP13	99th / SEEP11
Rhodobacter	38th / SEEP9	432nd / SEEP1
Rhodovulum	48th / SEEP1	204th / SEEP13
Shewanellaceae	29th / SEEP13	116th / SEEP1
Sulfuricurvaceae	*(Top Rank in All)*	
Sulfurospirillaceae	8th / SEEP11	26th / SEEP9
Sulfurovumaceae	3rd / SEEP11	11th / SEEP13
Syntrophaceae	22nd / SEEP9	30th / SEEP3
Wolbachia	15th / SEEP1	53rd / SEEP9

Table S6. Classifications of 18S clone library sequences from SEEP3, ordered by number of sequences across grouped relatives. Common names are given for related taxa.

Taxon	# of Clones	Common Affiliation
Thalassiosirales	28	Diatoms
Cymatosirales	8	
Gymnodiniales	26	Dinoflagellates
Dynophyceae	4	
Syndiniales	2	
Eugregarinorida	10	Parasitic protozoa
Unclassified Coccidia	1	
Archigregarinorida	1	
Laminariales	5	Kelp
Prasinophyceae	3	Green algae
Strombidiidae	2	Heterotrophic protozoa
Unclassified Cercozoa	1	
Eurotiales	1	Green/blue molds

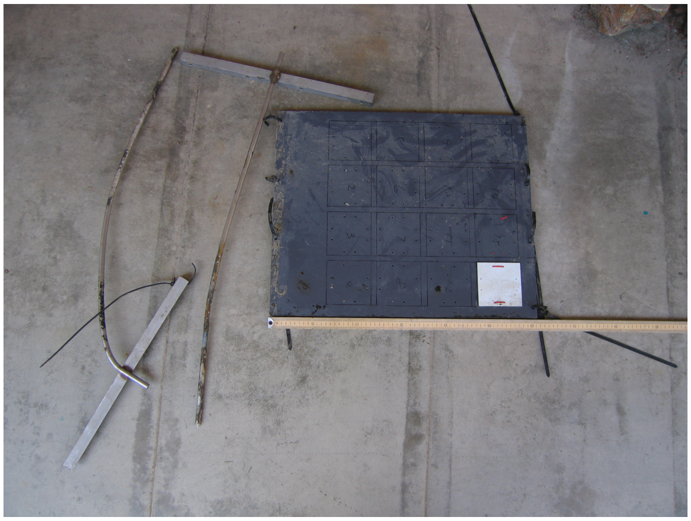

Figure S1. Benthic growth device retrieved after a large offshore storm in April 2007. Wave action was sufficient to badly damage the rebar used to secure the device at Shane Seep, dislodging the device and ending the SEEP1–14 series. Large PVC board (grey) is 60cm wide; small plate (white) is 10cm wide.

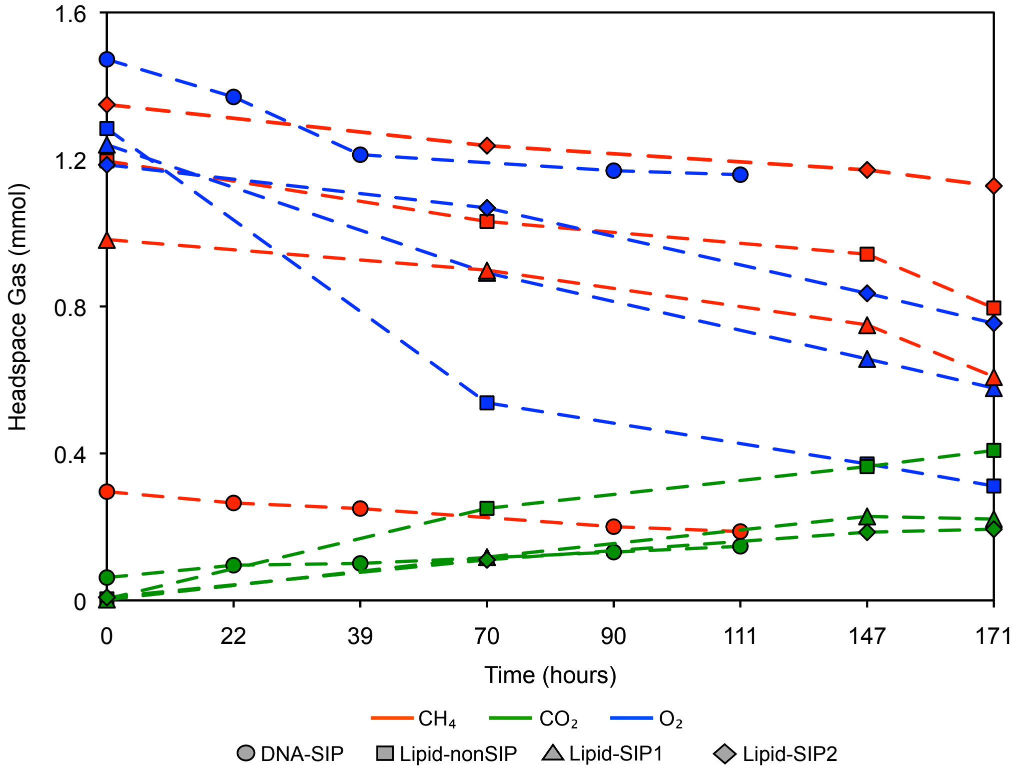

Figure S2. Changes in headspace gas composition during SIP ($^{13}CH_4$) incubations with microbial mats harvested from Shane Seep.

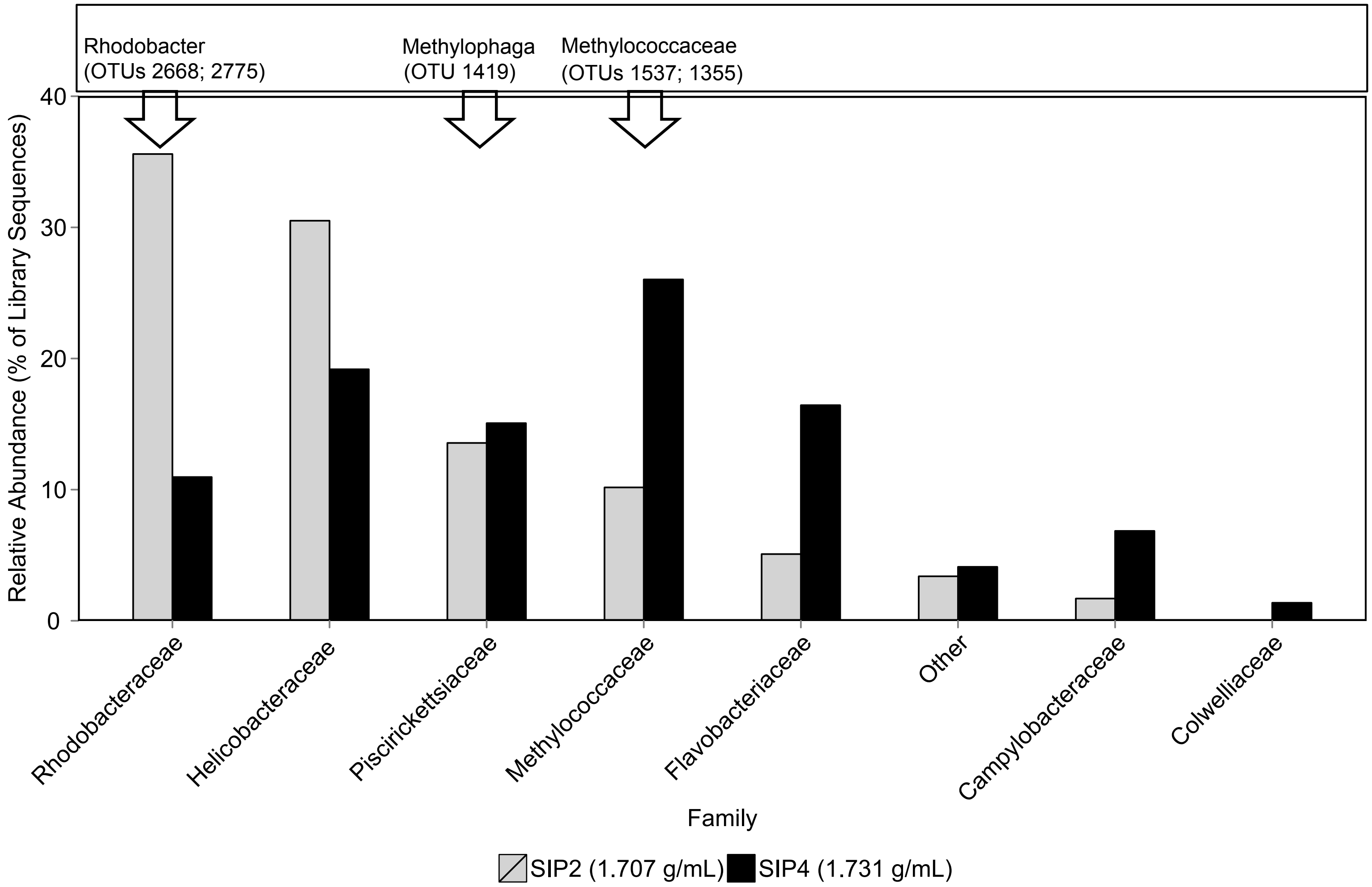

Figure S3. Family-level composition of SIP2 (59 sequences) and SIP4 (73 sequences) clone libraries, as determined by the RDP SeqMatch tool. Notable similarities between clone library results and PhyloChip results are marked with arrows and the corresponding PhyloChip OTUs. PhyloChip OTUs within Sulfurovum and Sulfuricurvum are most closely related to the family Helicobacteraceae.

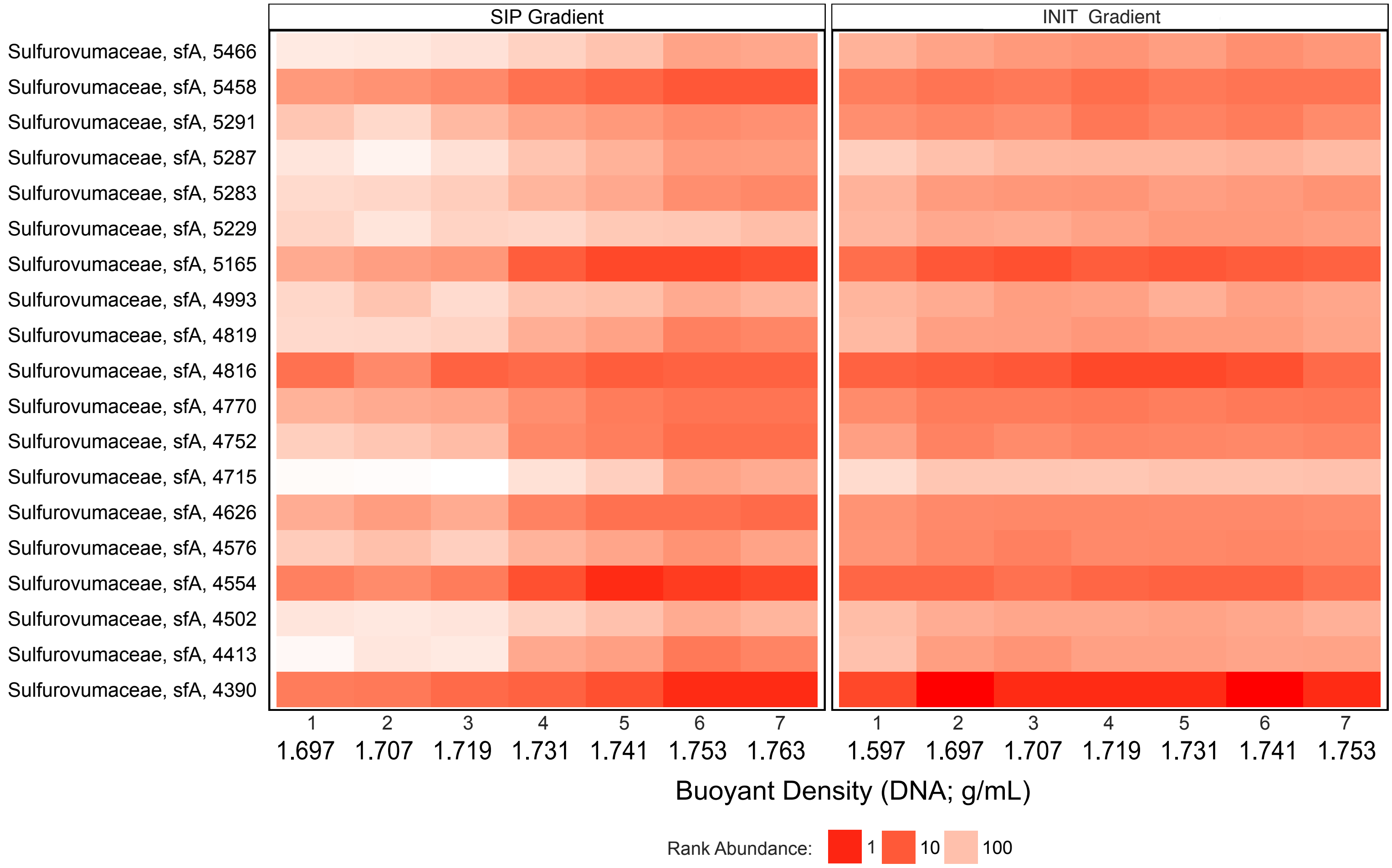

Figure S4. Heatmap of ranked abundances from OTUs belonging to *Sulfurovum* subfamily-A in the SIP (left) and INIT (right) density gradients. Fractions 1–7 from each gradient are labeled with their buoyant densities (g/mL).

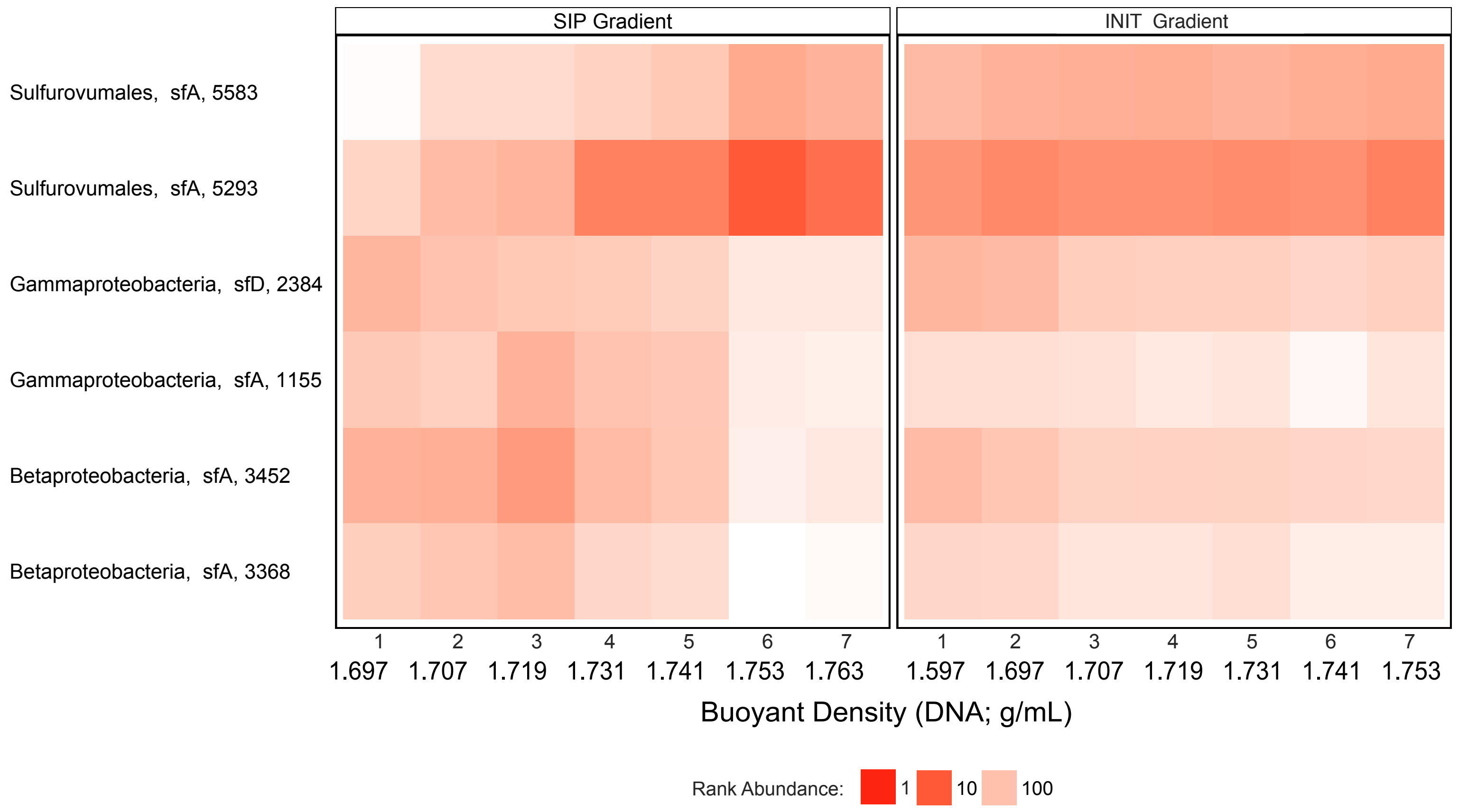

Figure S5. Heatmap of ranked abundances from unclassified OTUs in the SIP (left) and INIT (right) density gradients. Fractions 1–7 from each gradient are labeled with their buoyant densities (g/mL).

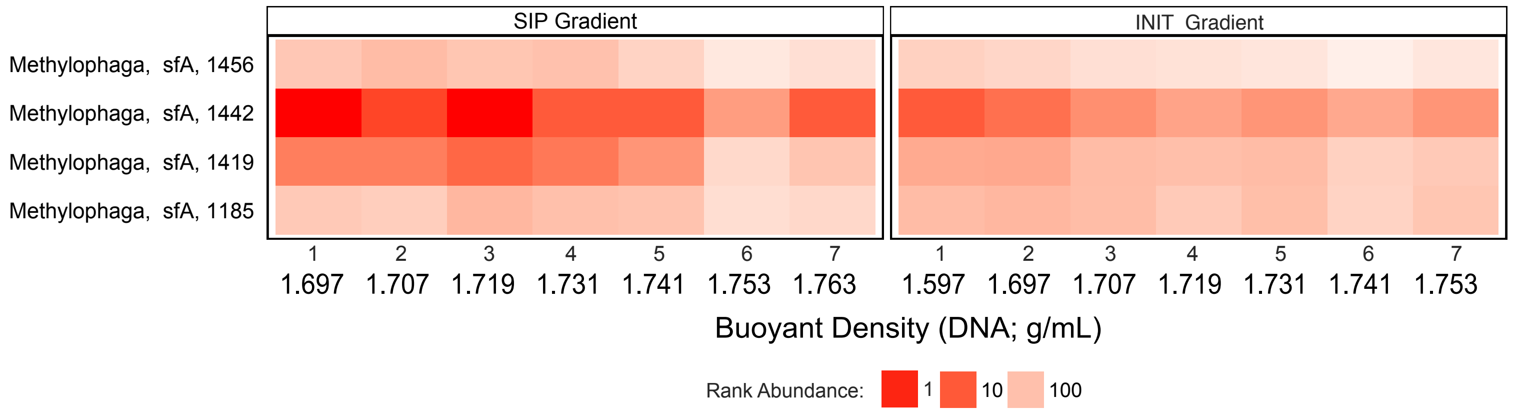

Figure S6. Heatmap of ranked abundances from OTUs belonging to *Methylophaga* subfamily-A in the SIP (left) and INIT (right) density gradients. Fractions 1–7 from each gradient are labeled with their buoyant densities (g/mL).

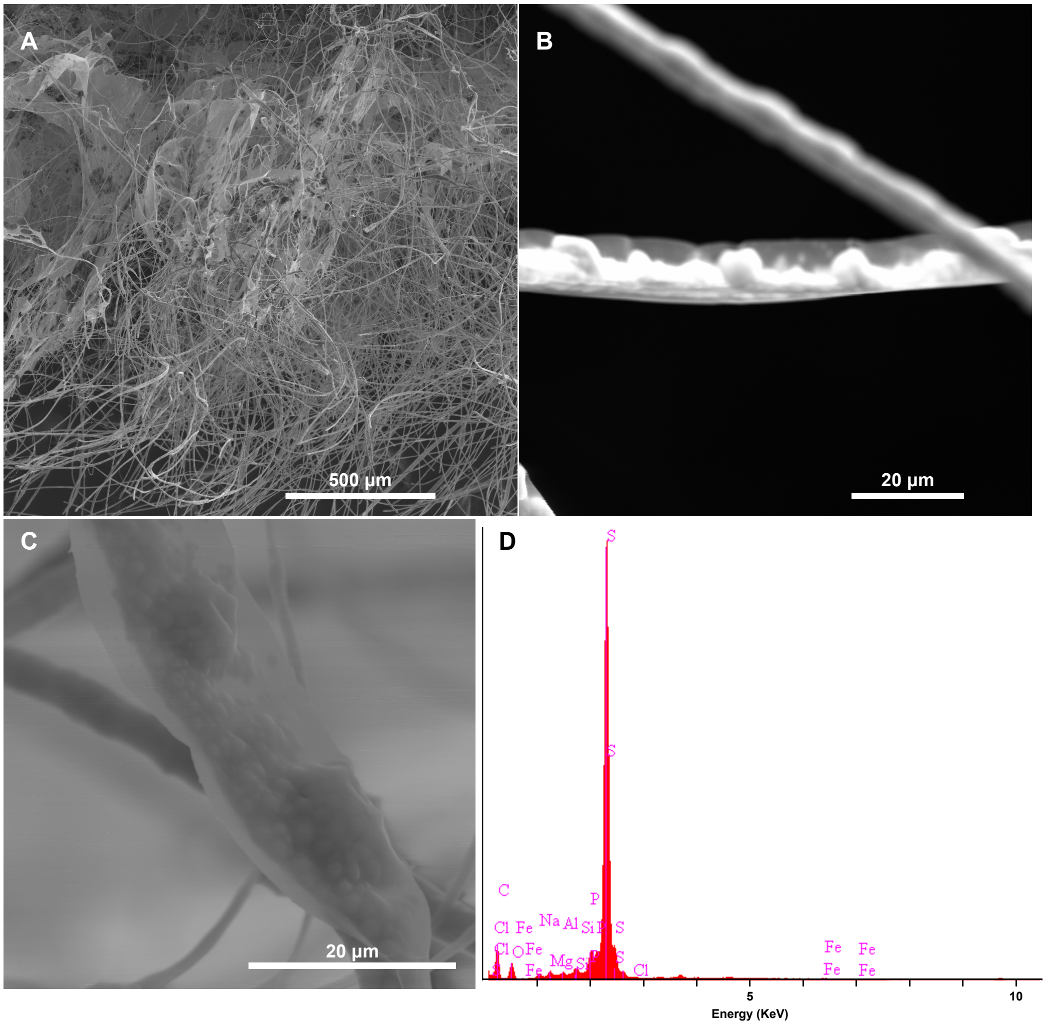

Figure S7. Scanning electron microscopy and X-ray spectrometry of a microbial mat sample. A – C. SEM images of a microbial mat and focused regions of a cell filament containing granule-like structures. D. Energy-dispersive X-ray spectromentry analysis of a cellular inclusion. Element labels are given for individual peaks.
